# Pennsylvania Hospital—*Surgical Wards*

**Published:** 1851-05

**Authors:** James J. Levick

**Affiliations:** Resident Physician; Pennsylvania Hospital


### CLINICAL REPORTS.

### PENNSYLVANIA HOSPITAL—*Surgical Wards.—Service of* DR. NORRIS.

*Cases discharged from November* 1, 1850, *to March* 1, 1851.

| | Cured. | By request. | Died. |
|---|---|---|---|
| Abscess, - - - - - - | 10 | 0 | 0 |
| Anthrax, - - - - - - | 1 | 0 | 0 |
| Boils, - - - - - - | 1 | 0 | 0 |
| Burns and scalds, - - - | 2 | 0 | 3 |
| Calculus, - - - - - - | 0 | 0 | 1* |
| Caries, - - - - - - | 2 | 0 | 0 |
| Cancer, - - - - - - | 1 | 2 | 0 |
| Cataract, - - - - - - | 1 | 0 | 0 |
| Concussion of spine, - - - | 1 | 0 | 0 |
| Conjunctivitis, (Chronic,) - - | 1 | 0 | 0 |
| Contusion, - - - - - | 23 | 0 | 0 |
| Fistula in ano, - - - - - | 1 | 0 | 0 |
| Fractures, simple, | | | |
| Arm, - - - - - - | 5 | 0 | 0 |
| Clavicle, - - - - - - | 5 | 0 | 0 |
| Forearm, - - - - - - | 5 | 0 | 0 |
| Jaw, - - - - - - | 1 | 0 | 0 |
| Leg, - - - - - - | 6 | 0 | 0 |
| Pelvis, - - - - - - | 1 | 0 | 0 |
| Thigh, - - - - - - | 12 | 1 | 0 |
| Fractures, compound, | | | 0 |
| Arm, - - - - - - | 1 | 0 | 0 |
| Finger, - - - - - - | 2 | 2 | 0 |
| Jaw, - - - - - - | 1 | 0 | 0 |
| Toes, - - - - - - | 2 | 0 | |
| Foot, - - - - - - | 1 | 0 | 0 |
| Forearm, - - - - - | 1 | 0 | 0 |
| Leg, - - - - - - | 3 | 0 | 1 |

* Scarlatina.

| | Cured. | By request. | Died. |
|---|---|---|---|
| Gangrene of foot, - - - - | 1 | 0 | 0 |
| Gonorrhœa, - - - - - | 2 | 3 | 0 |
| Hæmorrhoids, - - - - - | 2 | 0 | 0 |
| Hydrocele, - - - - - | 3 | 1 | 0 |
| Inflamed arm, - - - - - | 1 | 0 | 0 |
| " hand, - - - - - | 1 | 0 | 0 |
| " knee, - - - - - | 1 | 0 | 0 |
| " leg, - - - - - | 1 | 0 | 0 |
| " breast, - - - - | 1 | 0 | 0 |
| " face, - - - - - | 1 | 0 | 0 |
| " eye, - - - - - | 1 | 0 | 0 |
| " finger, - - - - | 1 | 0 | 0 |
| " bladder, - - - - | 0 | 1 | 0 |
| Inverted toe nail, - - - - | 1 | 0 | 0 |
| Iritis, - - - - - - - | 1 | 0 | 0 |
| Luxation, - - - - - - - | 5 | 1 | 0 |
| Necrosis, - - - - - - - | 1 | 0 | 0 |
| Moveable cartilage in knee joint, | 1 | 0 | 0 |
| Paronychia, - - - - - | 4 | 0 | 0 |
| Retention of urine, - - - - | 0 | 1 | 0 |
| Sprain, - - - - - - - | 1 | 0 | 0 |
| Staphyloma, - - - - - | 1 | 0 | 0 |
| Stricture, - - - - - - - | 1 | 0 | 0 |
| Syphilis, - - - - - - - | 22 | 8 | 0 |
| Tumor of abdomen, - - - - | 0 | 1 | 0 |
| Ulcers, - - - - - - - | 10 | 4 | 0 |
| Wounds, | | | |
| Gunshot, - - - - - | 6 | 0 | 0 |
| Wounds of abdomen, - - - | 2 | 0 | 1 |
| " arm, - - - - | 4 | 0 | 0 |
| " eye, - - - - | 2 | 0 | 0 |
| " finger, - - - - | 0 | 1 | 0 |
| " foot, - - - - | 1 | 0 | 0 |
| " scalp, - - - - | 11 | 0 | 0 |
| " tongue, - - - - | 1 | 0 | 0 |
| " throat, - - - - | 1 | 0 | 0 |

Note.—I am indebted to the librarian for the above table.—J. J. L.

The following pages, though possessing neither special interest nor novelty, are taken from some notes made during the winter.

*Abscess.*—Under this head are included several cases of mammary abscess, occurring in nursing women. The writer is not aware that in the treatment of this distressing affection, there is anything peculiar to this hospital. The plan of encompassing the gland, after the acute inflammation has subsided, with broad straps of adhesive plaster, applied from its base, is generally adopted, with, it is believed, much benefit, not only by the support given to the depending breast, but in promoting by its compression, the filling up of sinuses and the discharge of pus from the part.

*Burns and Scalds.*—A large number of burns and scalds have been under care since last report. These have been treated, as mentioned in the 12th No. of the *Examiner*, 1849, varied to meet particular indications.

A distressing case of burn from sulphuric acid, is now under care. The patient, a robust laborer aged 26, was admitted January 19th, 1851, a pitcher full of oil of vitriol having, fifteen hours before, been thrown at his face, by an Irish girl, in revenge for some real or imaginary insult.

The forehead and upper half of the face, the eye-lids and both scleroticæ, were completely cauterized, presenting the appearance of moistened kid leather. Through the partially opaque corneæ the pupils were seen, dilated but immoveable, and were subsequently found entirely insensible to the light, the nerve having been paralysed by the injury. Though complaining somewhat of pain, the man seemed to suffer most from fear of blindness, having been unable to see since the receipt of the injury.

The usual application of lint wetted with lime water and sweet oil was made to the face, and the solution of sulphate of morphia in tea-spoonful doses four times daily was given.

On the fourth day, sloughing of the eye-balls was found to be taking place ; the linimentum calcis was laid aside, and slippery elm mucilage during the day, and the poultice at night, were substituted. Continued the use of the anodyne and a good diet. 24th. Great restlessness and some delirium. 25th. Succeeded in leaving his bed while sweating profusely, and wandered about his room. 27th. To-day has pneumonia of the right lung, slightly involving the left lung—pulse frequent and feeble. ℞. Hyd. chlo. mit. gr. ½ opii. pulv. gr. ¼ q. s. h.

28th. Percussion dull over the right lung, crepitant rale heard throughout the left lung—loud tubal respiration—says he is unable to breathe—pulse 120 and very feeble. Omit the calomel and opium and substitute ammon carb. gr. v., syr. senegæ f. ss. every two hours—brandy punch, wine-glassful every three hours.

On the 30th, the patient had improved, and from this time the pneumonia symptoms gradually disappeared. The separation of the slough from the forehead was followed by copious hemorrhage which could only be arrested by tying the temporal artery.

Pus, and destroyed tissues, continued to be discharged from the eye-balls for a length of time. On the 10th of February, he was permitted to sit up;—total destruction of both eyes had now taken place, and a large ulcerated surface of the face, forehead and eye-lids remained. Cicatrization of the ulcers has gradually taken place, and the man is now able to walk abroad, and will probably be soon discharged.

*Dislocation of the (tarsal) scaphoid bone.*—This somewhat singular accident happened to a large, but pale and sickly man, aged 40, who was brought to the hospital, February 13, 1850. The history was imperfect, but so far as could be learned, the patient, while in the delirium of fever, had leaped from a window about twenty feet high, alighting on "the ball" of the foot. There was a very marked prominence on the upper surface of the foot immediately posterior to the articulation of the first metatarsal bone, and the man complained of pain on attempting any movement of the foot. The integument was tense, but there was no external wound.

An assistant having been directed to secure the leg, reduction was effected by placing the left hand on the foot immediately in advance of the leg, while the right hand firmly grasped the extremity of the metatarsal bones at the same time that the thumb was pressing back the prominent bone. Strong traction having been made, the bone suddenly yielded, slipped into its place with a snap, the patient screamed with pain, and the deformity at once disappeared. A figure-of-eight roller was applied over the foot, and the man closely watched. At the end of twelve hours this was removed, but no increase of swelling existing, it was re-applied. The man remained in the house several weeks under treatment for his febrile affection, but no unpleasant result followed the injury to the foot. At the time of his dismissal he walked with a cane without difficulty.

*Paronychia.*—Persons with this affection generally do not apply for aid at the hospital until matter has freely formed, has travelled beneath the fascia, destroyed the periosteum, and produced the death of the bone. Scarcely a week passes that this last result of the neglect of a free opening is not seen. It is believed that this opening can hardly be made too early, for though but little pus may have formed, yet the local depletion thus obtained will prove of benefit. Of whitlows and boils during the summer, there were so many cases as almost to give the affections the character of an epidemic.

*Ulcers.*—Although most of the cases under care are those of acute disease, it is impossible to avoid receiving a few of the kind here mentioned. The treatment of these, more especially of ulcers of the leg, is so simple as almost to be reducible to a single formula.

After he has been thoroughly cleansed by a warm bath, the patient is put to bed, the leg placed in a fracture box and slightly elevated. Over the ulcer and adjacent parts, a large but light flax-seed meal poultice covered with oiled silk is laid, to be renewed every twelve hours. If the tongue be coated and the bowels constipated, five or six grains of blue mass, followed by castor oil, are given.

For the first day or two, little is effected by the poultice, but the removal of the dirt and hardened discharge which have been allowed to accumulate, and which water alone will scarcely remove. If the ulcer be covered by a slough, the poultice is continued until this has separated; if on the other hand it presents a raw, jagged, indolent, unhealthy appearance, the stick of lunar caustic is thoroughly applied over the whole surface of the sore. In either case, after a few days, the slough separates leaving a healthy granulating surface. The poultice is now laid aside, and lint wetted with lime-water or cold water, is substituted; this is kept wet; if protected by oiled silk it is changed twice, or at least once in twenty-four hours. Once in twenty-four hours the ulcer and parts adjacent are thoroughly washed by a soft sponge with water and castile soap; and, to avoid any danger of contagion, each patient is provided with basin, soap and sponge, to be used for him alone.

If, as is very often the case, the patient should be anemic, or there should exist any vice of constitution, the syrup of the iodide of iron, from five to fifteen drops, or a pill containing of Vallet's proto-carb. of iron gr. iij, sulphate of quinia gr. iij, thrice daily is prescribed. In cases of syphilitic or scrofulous origin the iodide of potassium or cod liver oil is respectively given. Cicatrization generally goes on steadily. If this be tardy or the granulations become flabby, they are lightly touched with the solid lunar caustic.

At this time pressure is generally made use of; either by means of a few adhesive straps applied in such a way as not to retain the discharge, or a roller alone is firmly placed over the entire limb and its dressing. When the ulcer has cicatrized, the roller is still retained, and the patient directed to wash the part clean with a little soap and water daily. When additional support is deemed necessary, straps of adhesive plaster as recommended by Baynton and Critchett, are applied to the entire limb.

This simple treatment is found sufficient for many unpromising cases. Occasional benefit is derived by alternating with the wet dressings, the cerates of carbonate of zinc, or sub-acetate of lead.

For a year past there has been but little erysipelas in the wards; during the month of February there were a few cases. It has seemed to the writer that in some cases of indolent ulcer, a mild attack of erysipelas rather promoted than retarded cicatrization. There has recently been a tendency to the formation of vesications, of the newly formed cuticle, containing an irritating serum. The evacuation of the fluid and the application of the water dressing has been generally found sufficient to arrest this.

*Wounds.—Wound of the throat.* A German tailor, aged 21 years, was admitted on the night of December 26, 1850, having a few hours before, while intoxicated, made an incised wound of the throat, with a sharp bread-knife. There was an external wound of between six and seven inches; and on examination it was found that he had cut through the thyro-hyoid membrane, shaved off the corners of the thyroid cartilage, and completely divided the lower part of the pharynx, laying bare on either side the carotid arteries, which could be seen for several inches pulsating in their places.

There was but little hemorrhage, yet the man was in a prostrate condition, from which he speedily reacted under the application of heat and sinapisms; the little oozing of blood being readily checked by dry lint.

A little water dropped in the mouth passed out at the opening in the throat, and was followed by a violent fit of coughing; the edges of the wound being retracted, when the man was on his back, to the width of four fingers.

On the following morning a secretion of mucus had taken place, which the patient endeavored in vain to bring to his mouth, and which was so copious as to render it necessary to use a sponge attached to a whalebone, to remove it. The treatment determined on and subsequently pursued was as follows: The patient was placed in such a position as most facilitated the escape of the secretion. A piece of gauze, and over it linen spread with cerate, was loosely laid over the wound. Twice daily a large gum catheter was introduced in the œsophagus through the opening in the throat, and barley water or thin arrow root injected. To moisten his mouth a wet linen cloth and small pieces of ice were used.

In three days' time granulations were found to be springing up, and contraction of the external wound had taken place. On the fifth day after the receipt of the injury, the patient was able to swallow small quantities of thin broth, and the catheter was dispensed with.

At the expiration of a fortnight, the secretion having very much diminished, a stout muslin cap with long tapes attached, was fitted to his head, the chin brought down to the chest, and the whole retained by attaching the tapes to the thigh. The wound speedily filled up; in four weeks time he was allowed small pieces of bread, which he swallowed without difficulty.

There now remain two slight points of ulceration, one of which is kept up by the necrosis of a portion of the cartilage, several small pieces of which have come away. The other, now about the size of a fine probe, connects with the larynx; but under the application of sulphate of copper, is closing. He now eats meat without difficulty. Although unable to articulate as loudly as before, he is rapidly regaining his voice.

In this case, the old fashioned method of retaining the divided parts by sutures was entirely avoided, experience having proved it, as is well known, both inefficient and injurious. Rest, position, the cap, and after a time, gentle traction with adhesive plaster, and the occasional touching of the ulcer with lunar caustic, were the means made use of, with a result highly satisfactory.

JAMES J. LEVICK, Resident Physician.

*Pennsylvania Hospital, March*, 1851.

